# Effectiveness and Safety of Adding Bevacizumab to Platinum-Based Chemotherapy as First-Line Treatment for Advanced Non-Small-Cell Lung Cancer: A Meta-Analysis

**DOI:** 10.3389/fmed.2021.616380

**Published:** 2021-06-30

**Authors:** Yi Liu, Hui-Min Li, Ran Wang

**Affiliations:** ^1^Department of Respiratory and Critical Care Medicine, The First Affiliated Hospital of Anhui Medical University, Hefei, China; ^2^Department of Orthopedics and Spine Surgery, The First Affiliated Hospital of Anhui Medical University, Hefei, China

**Keywords:** bevacizumab, OS, PFS, ORR, non-small-cell lung cancer

## Abstract

**Background and Objective:** Previous studies have evaluated the efficacy (OS, overall survival; PFS, progression-free survival; ORR, objective response rate) and adverse events of bevacizumab combined with platinum-based chemotherapy in first-line treatment of advanced non-small-cell lung cancer (NSCLC) compared with chemotherapy alone. However, the results were inconsistent.

**Methods:** We conducted a comprehensive search of PubMed, EMBASE, and Cochrane Library for potentially eligible articles. The outcomes were evaluated in terms of risk ratio (RR) or hazard ratio (HR) and the associated 95% confidence intervals (CIs). Meta-analysis was performed using the Stata 12.0 software, and subgroup analyses were performed based on the treatment and bevacizumab dose.

**Results:** Six randomized controlled trials with 2,465 patients were included in this meta-analysis. The results demonstrated that bevacizumab significantly increased OS (HR = 0.87, 95% CI 0.79–0.96), extended PFS (HR = 0.65, 95% CI 0.54–0.77), and increased ORR (ES = 0.40, 95% CI 0.31–0.48) when added to first-line platinum-based chemotherapy in patients with advanced NSCLC. Subgroup analyses showed that only the higher dose (15 mg/kg) of bevacizumab plus carboplatin–paclitaxel significantly extended the OS and PFS, but both 7.5 mg/kg and 15 mg/kg of bevacizumab improved ORR. However, both 7.5 mg/kg and 15 mg/kg of bevacizumab could only increase PFS and ORR, but not extend OS, when added to cisplatin–gemcitabine. Bevacizumab significantly increased the risk of grade ≥3 events of febrile neutropenia, haemorrhagic events, hypertension, leukopenia, neutropenia, and proteinuria.

**Conclusion:** Bevacizumab significantly increases OS, PFS, and ORR when added to first-line platinum-based chemotherapy in patients with advanced NSCLC, with no new safety signals found. Moreover, bevacizumab (15 mg/kg) plus carboplatin–paclitaxel is a better alternative in increasing OS to carboplatin–paclitaxel and bevacizumab (7.5 mg/kg and 15 mg/kg) plus cisplatin–gemcitabine.

## Introduction

Nearly one million people die of lung cancer every year, and non-small-cell lung cancer (NSCLC) accounts for 85% of all lung cancer (1, 2). Besides, more often, the NSCLC has advanced at the time of initial diagnosis (3). The recommended standard platinum chemotherapy regimen has limited clinical efficacy for the first-line treatment of advanced NSCLC (4–6), and thus more effective regimens are needed.

Bevacizumab is a kind of human monoclonal antibody (7), which can specifically target vascular endothelial growth factor, inhibit angiogenesis, and thus inhibit tumor growth and survival. Previous studies have shown that the addition of bevacizumab to carboplatin–paclitaxel (BCP) could significantly prolong overall survival (OS), progression-free survival (PFS), and improve the objective response rate (ORR), vs. chemotherapy alone (8–10). However, other studies showed that BCP did not significantly extend OS (11–13). In addition, some studies also showed that the addition of bevacizumab to cisplatin–gemcitabine (BCG) significantly extended PFS and improved the ORR vs. placebo when added to CG but did not significantly extend OS (14). This may partly be due to different sample sizes, different races, and other confounding factors.

To overcome the limitations of individual studies, three meta-analysis studies were published in 2010, 2011, and 2013 (2, 15, 16). Yang et al. (15) found that low-dose bevacizumab significantly increased PFS in patients with non-resectable NSCLC, while high-dose bevacizumab increased 2-year OS, extended PFS, and increased ORR. The team of Botrel reported that the higher dose of bevacizumab significantly extended the overall PFS, but the results of extended OS were inconclusive (16). However, Soria et al. (2) found that bevacizumab significantly prolonged OS and PFS when added to first-line platinum-based chemotherapy in patients with advanced NSCLC. Since then, there are new studies that reported the efficacy of bevacizumab-based first-line treatment of advanced NSCLC (10, 17). Therefore, the purpose of this study was to further evaluate the efficacy (OS, PFS, ORR) and adverse events (AEs) of bevacizumab combined with platinum-based chemotherapy in first-line treatment of advanced NSCLC compared with chemotherapy alone.

## Methods

This meta-analysis was performed according to the Cochrane Handbook for Systematic Reviews of Interventions (18) and presented based on the Preferred Reporting Items for Systematic Reviews and Meta-analyses (PRISMA) guidelines (19).

### Search Trials

We conducted an exhaustive literature search of PubMed, EMBASE, and Cochrane Library for randomized controlled trials (RCTs) that compared bevacizumab plus platinum-based chemotherapy to chemotherapy alone for treating patients suffering inoperable locally advanced (stage IIIB), recurrent, or metastatic (stage IV) NSCLC as first-line therapy. The search terms included “bevacizumab,” “avastin,” “lung neoplasms,” “neoplasms pulmonary,” “pulmonary cancers,” “cancer of lung,” and “randomized controlled trial.” The detailed search strategy is reported in Supplementary Table 1. We did not limit the languages or publication date. The literature search was last updated on August 20, 2020. Two reviewers (H.-M. L. and Y. L.) independently searched all the titles, and the abstracts and references of relevant studies were also reviewed for additional worthy literatures. In case of uncertainty, full-text articles were obtained, and any divergence was resolved by the agreement of the reviewers.

### Inclusion and Exclusion Criteria

Trials were selected and excluded based on the PICOS principle, as follows (1) Participants: the study population consisted of patients with locally advanced, metastatic, or recurrent NSCLC and an Eastern Cooperative Oncology Group performance status of 0 to 1. Patients who have previously undergone chemotherapy, immunotherapy, or other therapies are appropriate, with the exception of those who have previously received anti-VEGF medication. Patients with lymphomas, tumors in other organs, or multiple lung tumors (i.e., patients with small cell or mixed histology) were removed. Trials with an unknown or important baseline difference between groups were also omitted. (2) Interventions: the intervention in the experimental group was bevacizumab plus platinum-based chemotherapy (BCG or BCP). (3) Comparisons: the intervention in the control group was chemotherapy alone (CG or CP). (4) Outcomes: studies qualified when at least one of the following outcomes was present: hazard ratios (HRs) and 95% CI for OS and PFS, ORR, AEs (anemia, febrile neutropenia, haemorrhagic events, hypertension, leukopenia, neuropathy, neutropenia, proteinuria, thrombosis, diarrhea, and abdominal pain). (5) Study design: RCTs were eligible. Cohort studies, case–control studies, case reports, retrospective studies, systematic reviews, and meta-analyses were excluded.

### Risk-of-Bias Assessments

The Cochrane risk-of-bias criteria (18) were used to independently assess the methodological quality of the included RCTs by two researchers (H.-M. L. and Y. L.), which included seven items on randomization sequence generation, allocation concealment, blinding of participants and personnel, blinding of outcome assessment, incomplete outcome data, selective reporting, and other biases. We defined other biases as the baseline characteristics of different intervention groups were different.

### Data Extraction

Two reviewers (H.-M. L. and Y. L.) independently performed data extraction from the qualified studies. Any disagreement was resolved by discussion or by consulting a third reviewer (R. W.). The indispensable study characteristics, namely, first author, publication year, treatments, doses, sample size, patient characteristics, experimental and control interventions, and outcomes, were extracted.

The outcome measures of interest consisted of HRs and 95% confidence interval (CI) for OS and PFS, ORR, and AEs (anemia, febrile neutropenia, haemorrhagic events, hypertension, leukopenia, neuropathy, neutropenia, proteinuria, thrombosis, diarrhea, and abdominal pain).

### Statistical Analysis

In this meta-analysis, we calculated the risk ratio (RR), with its 95% CI for ORR and risk of AEs, and the HR and its 95% CI for OS and PFS.

Statistical heterogeneity (20–24) was assessed using the I^2^ and chi-squared tests at a significance level of *P* < 0.05. If heterogeneities existed, one of the following techniques was used to attempt to explain such heterogeneities: (1) random-effect model for meta-analysis, (2) subgroup analyses, and (3) sensitivity analyses. Based on the treatment and bevacizumab dose, subgroup analyses of the OS, ORR, and PFS were performed. Besides, stratified analyses for the efficacy (OS and PFS) were also taken into account the patient characteristics (dose, sex, age, stage, race, bodyweight loss, smoking status, and histology). Moreover, we assessed the publication bias by performing the funnel plot test. Statistical analysis was performed using Stata 12.0. All tests were two-tailed, and *P* < 0.05 was considered statistically significant.

## Results

### Study Search

**Figure 1:**
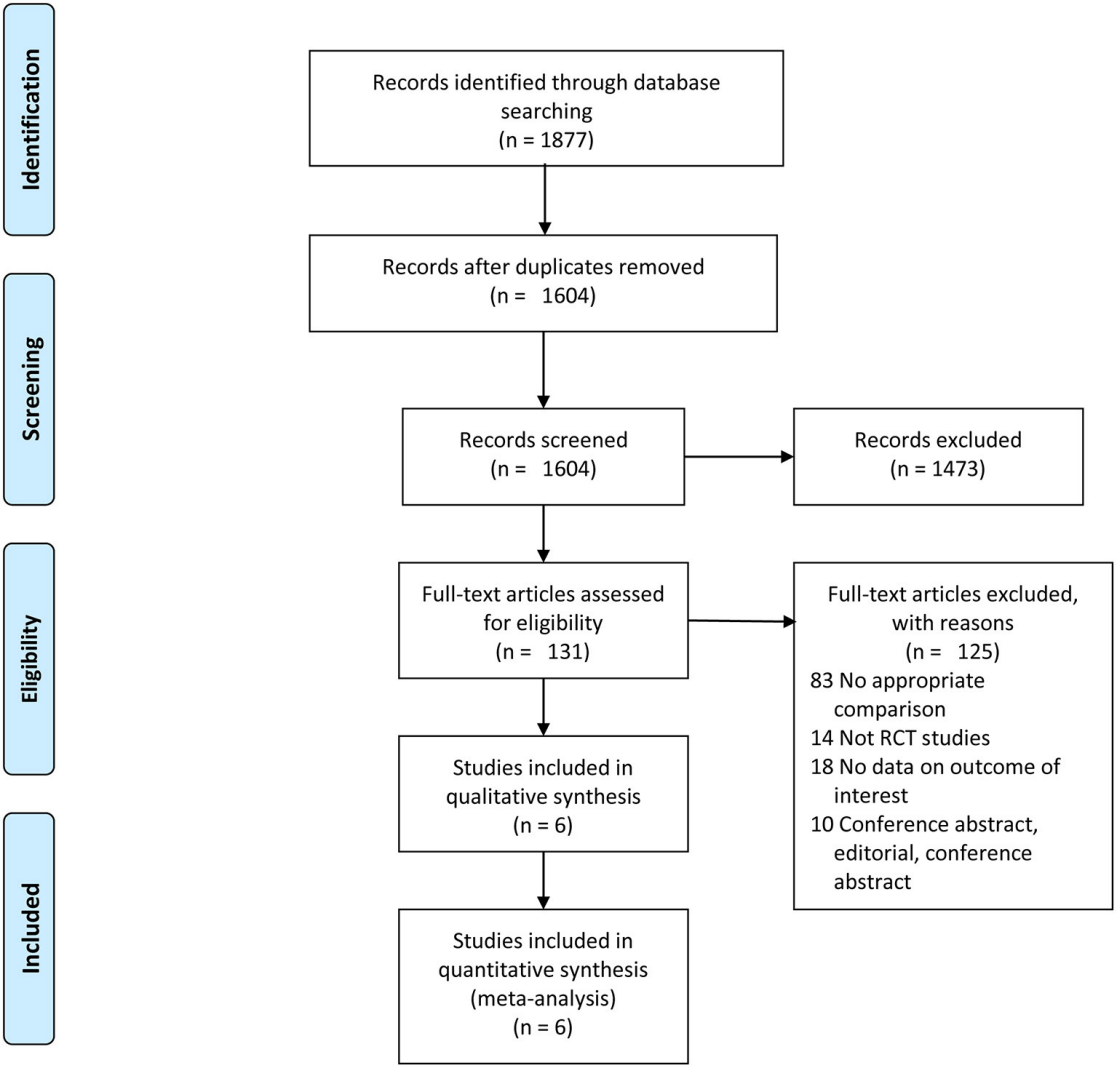
Flow diagram of the study selection.

A summary of the study selection process is shown in Figure 1. A total of 1,877 relevant studies were inspected *via* electronic search. A total of 273 studies were excluded because they were duplicates. After assessing the titles and abstracts, 1,473 studies were eliminated, because they did not meet the eligibility criteria. After verifying the full text of the remaining 131 studies, 6 RCTs (8, 10, 13, 14, 25) with 2,465 patients were finally included in this meta-analysis.

### Study Characteristics

Table 1 summarizes the main characteristics of the studies included. The baseline information on the five studies of the two groups was balanced and comparable (8, 10, 13, 14). In the six identified RCTs, all studies were from a multicenter, and treatment was performed in 3-week cycles for up to six cycles, or until disease progression, or unacceptable toxicity. Among the six studies, three studies evaluated two doses of bevacizumab (7.5 mg/kg and 15 mg/kg) (14, 25); other studies evaluated only one bevacizumab dose (15 mg/kg). Besides, two studies (14) compared bevacizumab plus cisplatin–gemcitabine with cisplatin–gemcitabine, and four studies (8, 10, 13, 25) compared bevacizumab plus cisplatin–gemcitabine with carboplatin–paclitaxel.

**Table 1:** Characteristics of the included studies.

**Study**	**Year**	**Design**	**Number of patients**		**Treatment**		**Stage**	**Outcomes**
			**Trial**	**Control**	**Trial**	**Control**		
Reck M, et al. (17)	2010	Multicenter, randomized, double-blind phase III study	696	347	CG+bevacizumab 7.5 mg/kg (arm1) or 15 mg/kg (arm2)	CG	Advanced or recurrent non-squamous NSCLC	OS
Reck M, et al. (14)	2009	Multicenter, randomized, double-blind phase III study	696	347	CG+bevacizumab 7.5 mg/kg (arm1) or 15 mg/kg (arm2)	CG	Advanced or recurrent non-squamous NSCLC	PFS, ORR, AEs
Zhou C, et al. (10)	2015	Multicenter, randomized, double-blind phase III study	138	138	CP+bevacizumab 15 mg/kg	CP	Advanced or recurrent non-squamous NSCLC	OS, PFS, ORR, AEs
Niho S, et al. (13)	2012	Multicenter, randomized, open-label, phase II study	121	59	CP+bevacizumab 15 mg/kg	CP	Advanced or recurrent non-squamous NSCLC	OS, PFS, ORR, AEs
Sandler A, et al. (8)	2006	Multicenter, randomized, open-label, phase III study	427	440	CP+bevacizumab 15 mg/kg	CP	Advanced or recurrent non-squamous NSCLC	OS, PFS, ORR, AEs
Johnson D.H, et al. (25)	2004	Multicenter, randomized, open-label, phase II study	67	32	CP+bevacizumab 7.5 mg/kg (arm1) or 15 mg/kg (arm2)	CP	Advanced or recurrent NSCLC	OS, PFS, ORR, AEs

*CG, cisplatin-gemcitabine; CP, carboplatin-paclitaxel; OS, overall survival; PFS, progression-free survival; ORR, objective response rate; AEs, adverse events; NSCLC, non-small cell lung cancer*.

### Risk of Bias in the Included Studies

**Figure 2:**
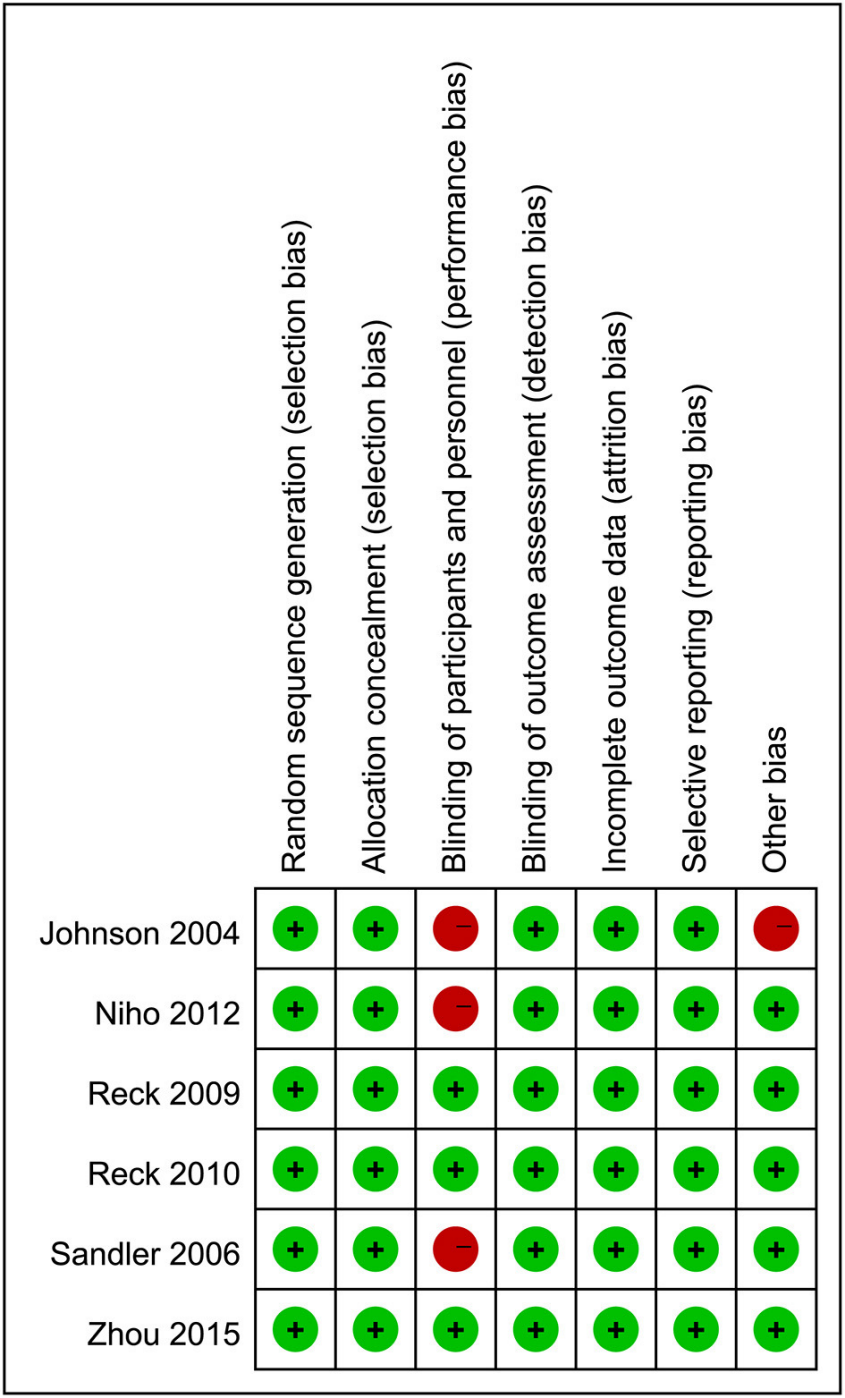
Risk of bias summary. + = low risk of bias; - = high risk of bias; ? = unclear risk of bias.

The risk of bias in the included studies is presented in Figure 2. All studies showed appropriate randomization and described the allocation concealment in detail. Three studies (10, 14) reported adequate blinding of participants and personnel. All studies reported adequate blinding of outcome assessment. In terms of incomplete outcome data, selective reporting, all studies were deemed to have a low risk of bias. For other bias, one study (25) was deemed to have a high risk of bias.

### OS

**Figure 3:**
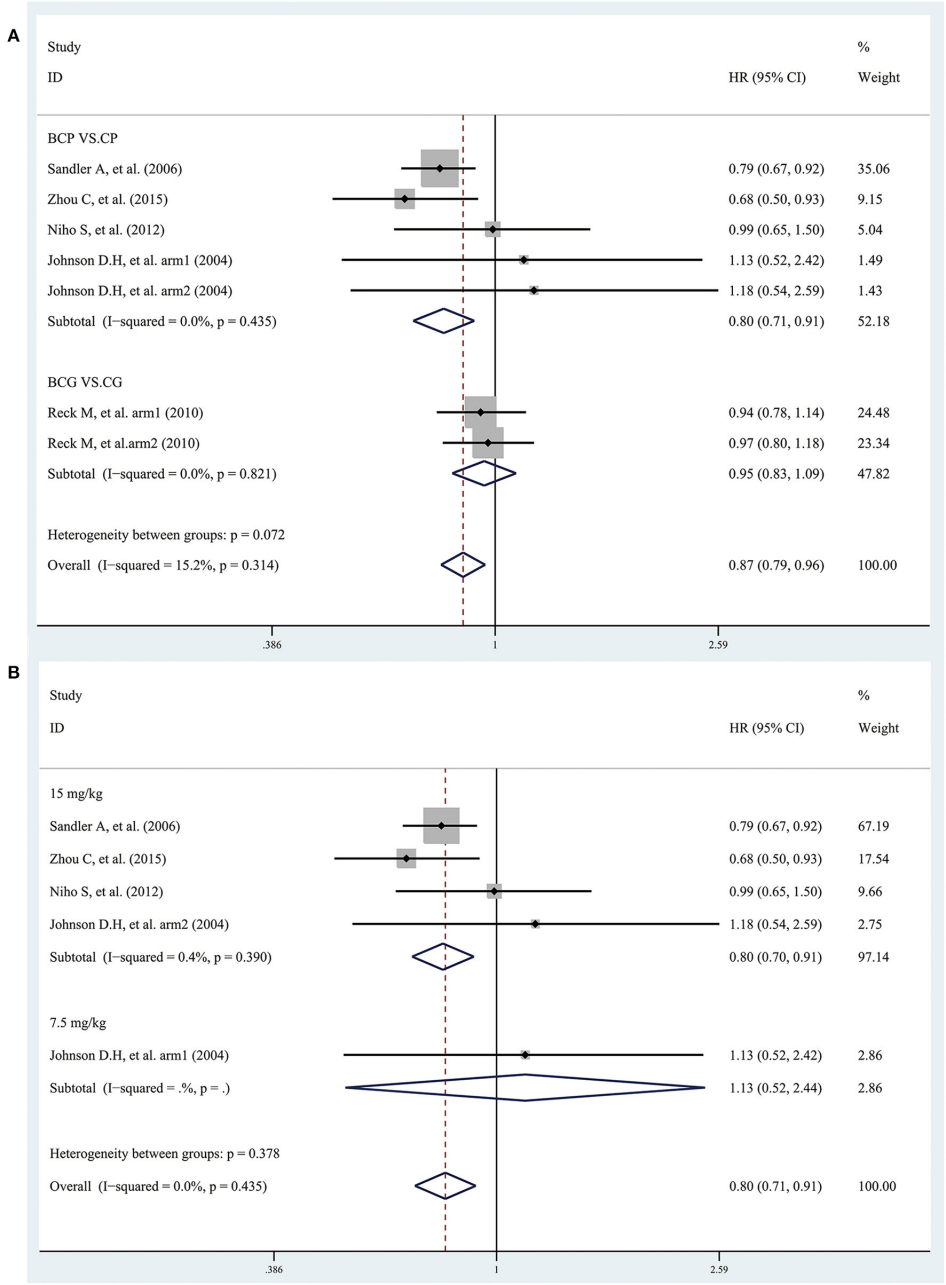
Forest plots of hazard ratios (HRs) for overall survival (OS) based on **(A)** treatment and **(B)** bevacizumab dose (7.5 mg/kg and 15 mg/kg) from randomized controlled trials of bevacizumab added to standard chemotherapy, compared with chemotherapy alone, as first-line therapy in patients with advanced NSCLC.

Five studies provided data on OS. The combined results showed that the overall OS was significantly longer in patients treated with bevacizumab plus platinum-based chemotherapy than with chemotherapy alone, with an estimated HR of 0.87 (95% CI 0.79–0.96; I^2^ = 15.2%, *P* = 0.314) (Figure 3A). Subgroup analyses based on treatment showed that the OS was significantly longer in patients treated with BCP than with CP, with an estimated HR of 0.80 (95% CI 0.71–0.91; I^2^ = 0.0%, *P* = 0.435). However, the combined results showed that the OS in patients treated with BCG was insignificantly different from with CG, with an estimated HR of 0.95 (95% CI 0.83–1.09; I^2^ = 0.0%, *P* = 0.821), and there was no significant difference between the two bevacizumab doses (Figure 3A). Besides, the combined results, stratified according to bevacizumab dose, showed that the overall OS was significantly longer only in patients treated with bevacizumab (15 mg/kg) plus CP than with CP, but not in patients treated with bevacizumab (7.5 mg/kg) (Figure 3B).

### PFS

**Figure 4:**
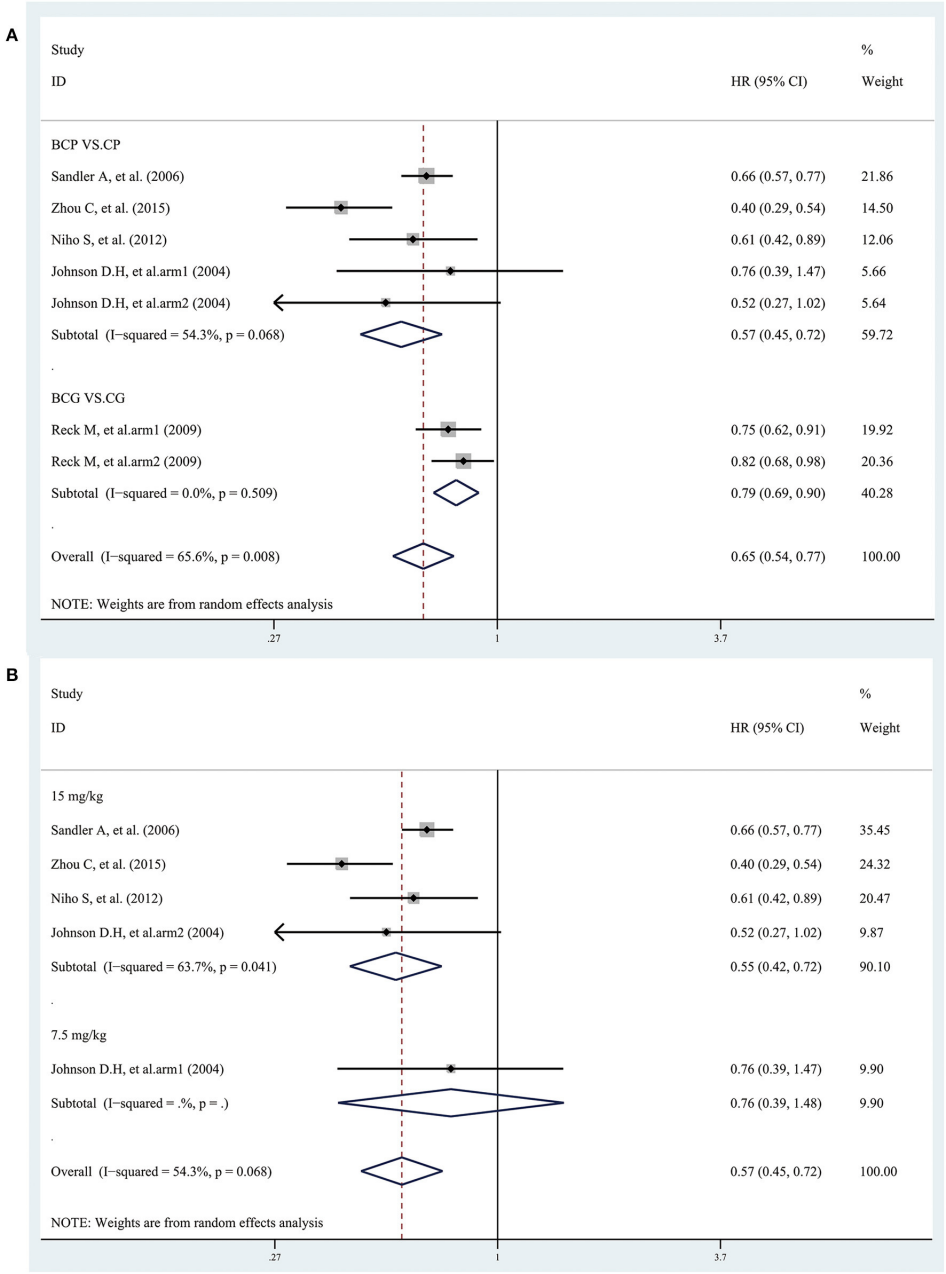
Forest plots of hazard ratios (HRs) for progression-free survival (PFS) based on **(A)** treatment and **(B)** bevacizumab dose (7.5 mg/kg and 15 mg/kg) from randomized controlled trials of bevacizumab added to standard chemotherapy, compared with chemotherapy alone, as first-line therapy in patients with advanced NSCLC.

Five studies provided data on PFS. The combined results showed that the overall PFS was significantly longer in patients treated with bevacizumab plus platinum-based chemotherapy than with chemotherapy alone, with an estimated HR of 0.65 (95% CI 0.54–0.77; I^2^ = 65.6%, *P* = 0.008) (Figure 4A). Subgroup analyses based on treatment showed that the PFS was significantly longer in patients treated with BCP or BCG than with CP or CG, with an estimated HR of 0.57 (95% CI 0.45–0.72; I^2^ = 54.3%, *P* = 0.068) and 0.79 (95% CI 0.69–0.90; I^2^ = 0.0%, *P* = 0.509), respectively (Figure 4A). Besides, the combined results, stratified according to bevacizumab dose, showed that the overall PFS was significantly longer only in patients treated with bevacizumab (15 mg/kg) plus CP than with CP, but not in patients treated with bevacizumab (7.5 mg/kg) (Figure 4B). However, the combined results showed that the overall PFS was significantly longer in patients treated with both bevacizumab (15 mg/kg) and bevacizumab (7.5 mg/kg) plus CG than with CG (Figure 4A).

### Interaction Between Patient Characteristics and OS and PFS

Bevacizumab showed a significantly greater treatment effect on OS in male patients (HR = 0.70, 95% CI 0.58–0.84), and in patients with age <65 years (HR = 0.68, 95% CI 0.57–0.81). Although there was a statistical difference between bevacizumab plus platinum-based chemotherapy and chemotherapy alone in race (white), smoking status (past), bodyweight loss (≤ 5%), and histology (adenocarcinoma), the results were only extracted from one study. There was no significant interaction between the treatment effect of OS and stage (Supplementary Table 2).

Bevacizumab showed a significantly greater treatment effect on PFS in patients with age <65 y (HR = 0.39, 95% CI 0.30–0.51), and in recurrent NSCLC patients (HR = 0.24, 95% CI 0.07–0.85). Although there was a statistical difference between bevacizumab plus platinum-based chemotherapy and chemotherapy alone in histology (large cell), the results were only extracted from one study. There was no significant interaction between the treatment effect of PFS and sex, smoking status (Supplementary Table 3).

### ORR

**Figure 5:**
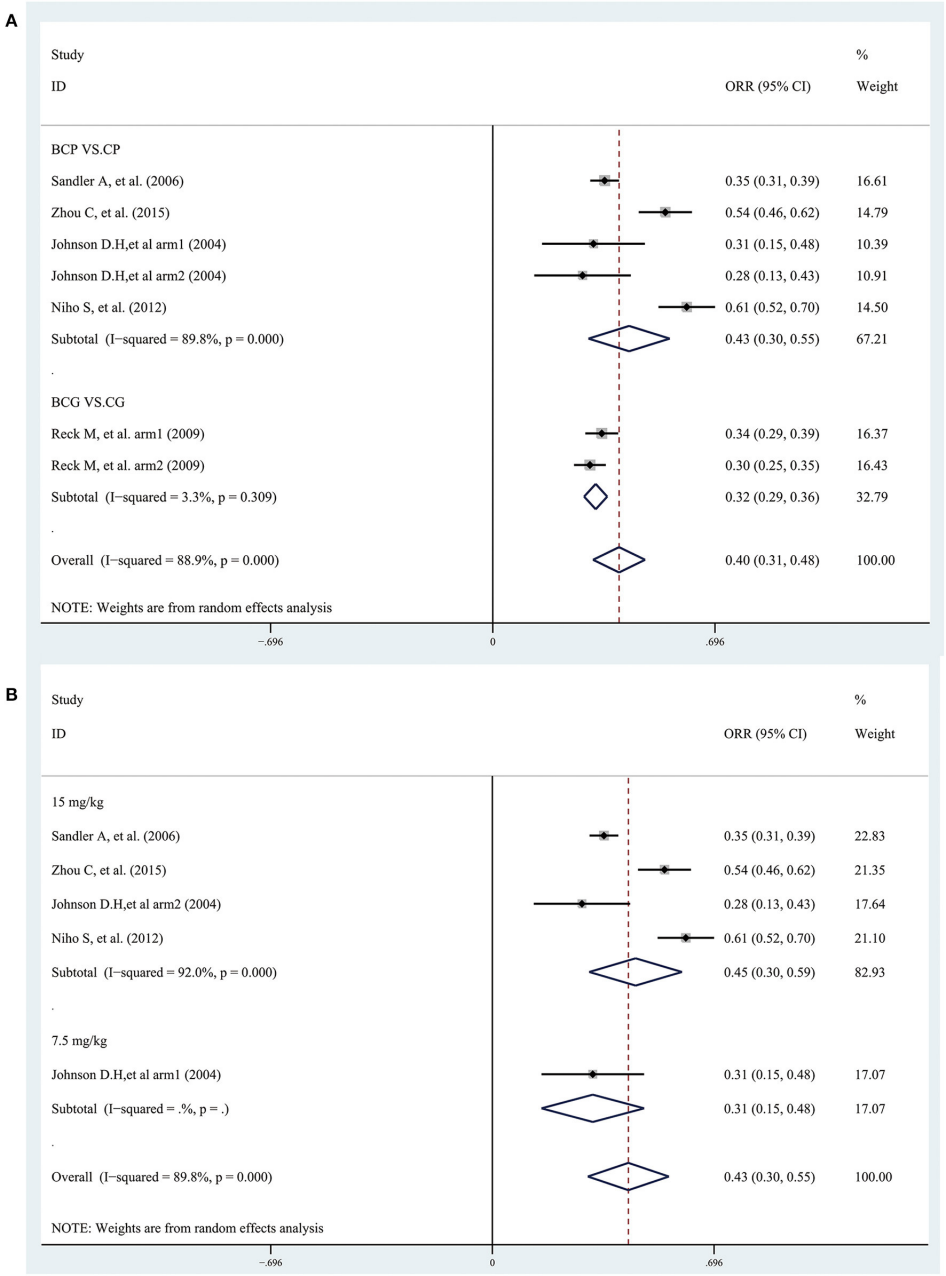
Forest plots of the objective response rate (ORR) based on **(A)** treatment and **(B)** bevacizumab dose (7.5 mg/kg and 15 mg/kg) from randomized controlled trials of bevacizumab added to standard chemotherapy, compared with chemotherapy alone, as first-line therapy in patients with advanced NSCLC.

Five studies provided data on ORR. The combined results showed that bevacizumab plus platinum-based chemotherapy significantly improved the overall ORR (ORR = 0.40, 95% CI 0.31–0.48; I^2^ = 88.9%, *P* = 0.000) (Figure 5A). Subgroup analyses based on treatment showed that the ORR was significantly improved in patients treated with BCP or BCG than with CP or CG, with an estimated ES of 0.43 (95% CI 0.30–0.55; I^2^ = 89.8%, *P* = 0.000) and 0.32 (95% CI 0.29–0.36; I^2^ = 3.3%, *P* = 0.309), respectively (Figure 5A). Besides, the combined results, stratified according to bevacizumab dose, showed that the ORR was significantly improved in patients treated with the combination of CP or CG plus bevacizumab (7.5 mg/kg and 15 mg/kg) than with CP or CG (Figures 5A,B).

### AEs

Data for AEs were available for five studies. According to the pooled analysis, bevacizumab significantly increased the risk of grade ≥3 events of febrile neutropenia (RR = 2.01; 95% CI 1.16–3.50), haemorrhagic events (RR = 3.20; 95% CI 1.82–5.63), hypertension (RR = 5.20; 95% CI 3.12–8.67), leukopenia (RR = 1.84; 95% CI 1.36–2.49), neutropenia (RR = 1.21; 95% CI 1.05–1.40), and proteinuria (RR = 10.09; 95% CI 2.88–35.39), compared with chemotherapy alone. However, there was no statistical difference in anemia, neuropathy, thrombosis, diarrhea, and abdominal pain between bevacizumab plus platinum-based chemotherapy and chemotherapy alone (Table 2). There was no significant heterogeneity between the studies in the overall toxicity analysis. Besides, subgroup analyses based on treatment showed no increased risk of proteinuria in patients treated with BCG compared to CG (RR = 5.96; 95% CI 0.72–49.32), and no increased risk of neutropenia in patients treated with BCP compared to CP (RR = 1.28; 95% CI 0.83–1.97).

**Table 2:** Subgroup analyses of AEs based on treatment.

**Category**		**No. of studies**	**Tests of association**				**Tests of heterogeneity**	
		**RR**	**95%CI**	***P*-value**	**Model**	**I^**2**^, %**	***P*-value**	
Anemia	Overall	4	0.81	0.62,1.07	0.144	F	0.0	0.566
	BCP vs. CP	2	0.57	0.28,1.18	0.129	F	31.5	0.227
	BCG vs. CG	2	0.87	0.65,1.17	0.361	F	0.0	0.992
Diarrhea and abdominal pain	Overall	3	1.18	0.36,3.82	0.783	F	46.0	0.157
	BCP vs. CP	3	1.18	0.36,3.82	0.783	F	46.0	0.157
Febrile neutropenia	Overall	3	2.01	1.16,3.50	**0.013**	F	0.0	0.595
	BCP vs. CP	3	2.01	1.16,3.50	**0.013**	F	0.0	0.595
Haemorrhagic events	Overall	4	3.20	1.82,5.63	**0.000**	F	0.0	0.508
	BCP vs. CP	2	6.31	2.05,19.36	**0.001**	F	0.0	0.872
	BCG vs. CG	2	2.32	1.19,4.51	**0.014**	F	0.0	0.996
Hypertension	Overall	7	5.20	3.12,8.67	**0.000**	F	0.0	0.447
	BCP vs. CP	5	5.67	2.58,12.46	**0.000**	F	28.9	0.229
	BCG vs. CG	2	4.86	2.49,9.52	**0.000**	F	0.0	0.672
Leukopenia	Overall	4	1.84	1.36,2.49	**0.000**	F	0.0	0.942
	BCP vs. CP	4	1.84	1.36,2.49	**0.000**	F	0.0	0.942
Neuropathy	Overall	3	1.98	0.50,7.78	0.327	F	0.0	0.377
	BCP vs. CP	3	1.98	0.50,7.78	0.327	F	0.0	0.377
Neutropenia	Overall	4	1.21	1.05,1.40	**0.000**	R	54.4	0.087
	BCP vs. CP	2	1.28	0.83,1.97	0.273	R	88.6	0.003
	BCG vs. CG	2	1.19	1.02,1.38	**0.023**	F	0.0	0.440
Proteinuria	Overall	5	10.09	2.88,35.39	**0.000**	F	0.0	0.647
	BCP vs. CP	3	12.58	2.61,60.57	**0.002**	F	2.1	0.360
	BCG vs. CG	2	5.96	0.72,49.32	0.098	F	0.0	0.613
Thrombosis	Overall	4	1.01	0.69,1.48	0.946	F	0.0	0.436
	BCP vs. CP	2	0.37	0.09,1.45	0.152	F	16.2	0.275
	BCG vs. CG	2	1.11	0.69,1.48	0.609	F	0.0	0.923

*R: random-effect model; F: fixed-effect model; RR, risk ratio; CI, confidence intervals. Bold values indicate P < 0.05. HR, hazard ratio; CI, confidence intervals. Bold values indicate P < 0.05*.

### Sensitivity Analyses and Publication Bias

**Figure 6:**
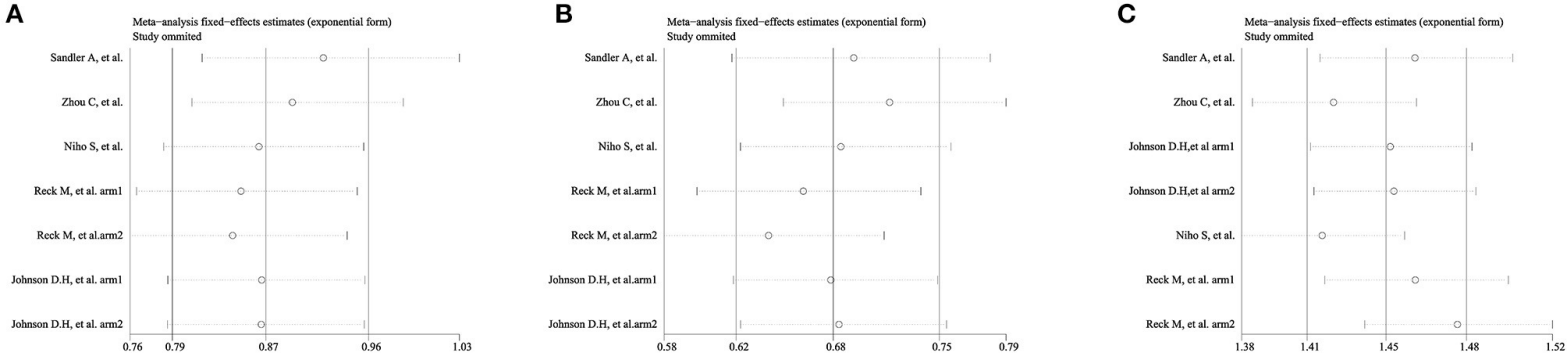
Sensitivity analysis of the **(A)** overall survival (OS), **(B)** progression-free survival (PFS), and **(C)** objective response rate (ORR).

**Figure 7:**
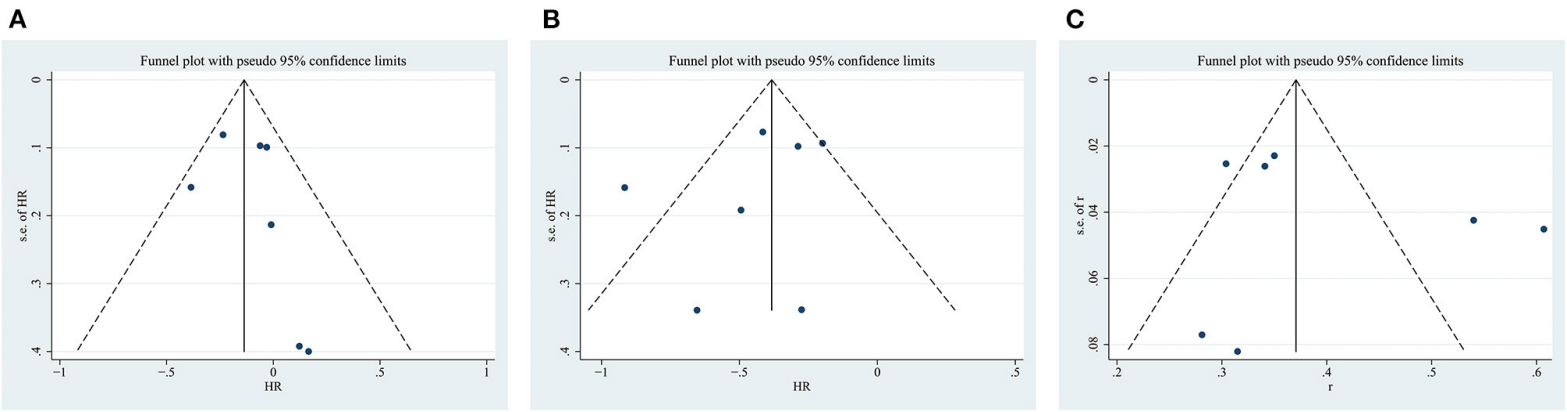
Funnel plot for the **(A)** overall survival (OS), **(B)** progression-free survival (PFS), and **(C)** objective response rate (ORR).

The sensitivity analysis omitted one study at a time and did not yield different results in terms of overall OS, PFS, and ORR (Figure 6). However, the funnel plot test showed that overall OS, PFS, and ORR had low publication bias (Figure 7).

## Discussion

The clinical benefits of bevacizumab have demonstrated a series of tumors, including colorectal cancer (26), metastatic breast (27), and renal cancer (28). In patients with advanced NSCLC, three phase III trials (8, 10, 17) demonstrated that bevacizumab significantly improved PFS after adding to standard first-line chemotherapy, but only two trials (8, 10) showed that OS was prolonged with the bevacizumab plus platinum-based chemotherapy. Therefore, this analysis pooled data from the Phase II and III trials to include more samples. Our analysis has several advantages: it took into account a larger sample, and all included studies were deemed as low risk of bias; its stratified analyses for the efficacy (OS, PFS, ORR) were based on treatment and bevacizumab dose; and it considered the effect of patient characteristics on OS and PFS.

According to this analysis, the use of bevacizumab plus platinum-based chemotherapy significantly prolonged OS, compared with chemotherapy alone. Subgroup analysis based on treatment and bevacizumab dose further showed that only 15 mg/kg bevacizumab plus CP could prolong the OS, but not 7.5 mg bevacizumab plus CP. Besides, neither 7.5 mg/kg nor 15 mg/kg of bevacizumab plus CG can prolong the OS. However, second- and third-line therapies (epidermal growth factor receptor tyrosine kinase inhibitor) have potentially confounding effects in the OS endpoint analysis (29). In the AVAiL (14) and JO 19907 (13), the number of patients in the chemotherapy alone group, who received subsequent chemotherapy, was slightly higher. In the AVF-0557g trial (25), nearly 60% of patients in the control group received cross-therapy with bevacizumab. In ECOG 4599 (8) and BEYOND (10), ~20 and 36% of patients in the bevacizumab group received second-line therapy with a tyrosine kinase inhibitor, respectively. Considering the effect of patient characteristics on OS, we found that bevacizumab showed a significantly greater treatment effect on OS in male patients, and in patients with age < 65 years, which were consistent with the previous studies (8, 10). However, other studies did not suggest a lower level of efficacy of bevacizumab in the elderly population (30, 31). This result may be explained by several factors including imbalances between the two groups in terms of known or unknown baseline prognostic factors (e.g., epidermal growth factor mutations), imbalances in the use of second - and third-line therapies, statistical chance.

The prolonged PFS benefit was observed in the bevacizumab plus platinum-based chemotherapy group. Subgroup analysis based on treatment and bevacizumab dose further showed that only 15 mg/kg bevacizumab plus CP could prolong the PFS, but not 7.5 mg bevacizumab plus CP. However, both 7.5 mg/kg and 15 mg/kg of bevacizumab plus CG can prolong the PFS. Many studies have demonstrated that the addition of bevacizumab to first-line platinum-based chemotherapy significantly improved PFS in patients with advanced NSCLC (8, 10, 13, 14, 25). Besides, both the Food and Drug Administration and the European Medicines Agency have accepted PFS as an effective measure of clinical benefit, especially when further treatment is expected to hinder testing of related OS benefits (17).

Consistent with the previous studies (8, 14, 25), the improvement of ORR was observed in the bevacizumab plus platinum-based chemotherapy group in the present study. Subgroup analysis based on treatment and bevacizumab dose further showed that both 7.5 mg/kg and 15 mg/kg of bevacizumab, plus CG or CP, can improve the ORR. This result further supports the hypothesis that drug delivery to the tumor could be improved by bevacizumab (26).

In the present study, bevacizumab significantly increased the risk of grade ≥3 events of febrile neutropenia, haemorrhagic events, hypertension, leukopenia, neutropenia, and proteinuria, compared with chemotherapy alone. The AEs of bevacizumab were consistent with the previous reports (2), and no new evident toxicity patterns have been found in the current analysis, supporting the well-established and controllable adverse event profile for bevacizumab (17). However, we cannot take dose effects into account due to limited data, as previous studies did (2, 16).

Meta-analysis is a powerful statistical method that provides a quantitative way to combine data from independent studies and examine and interpret heterogeneity. In 2010, Yang et al. (15) carried out the first meta-analysis to investigate the effectiveness and safety of bevacizumab for unresectable NSCLC. They pooled data from four studies, included 2,101 patients, and stratified their analysis according to bevacizumab dose. The results of this study suggested that low-dose bevacizumab significantly increased PFS in patients with non-resectable NSCLC, while high-dose bevacizumab increased the 2-year OS, extended the PFS, and increased the ORR. In 2011, Botrel et al. (16) found that the higher dose of bevacizumab significantly extended the overall PFS, but the results of the extended OS were inconclusive, which included 2,200 subjects. In 2013, the team of Soria reported that bevacizumab significantly prolonged OS and PFS when added to first-line platinum-based chemotherapy in patients with advanced NSCLC, which included 2,194 subjects. In our meta-analysis, a total of 2,465 subjects were finally included. Our result demonstrated that bevacizumab significantly increased the OS, extended the PFS, and increased the ORR when added to first-line platinum-based chemotherapy in patients with advanced NSCLC. Considering the effect of different platinum-based chemotherapies and bevacizumab doses on our meta-analysis results, we further performed subgroup analyses based on treatment and bevacizumab dose. Stratification analysis showed that only the higher dose (15 mg/kg) of bevacizumab plus CP significantly extended the OS, and PFS, but both 7.5 mg/kg and 15 mg/kg of bevacizumab improved the ORR. However, both 7.5 mg/kg and 15 mg/kg of bevacizumab could only increase the PFS and ORR, but not extend the OS, when added to CG.

There are some limitations in this meta-analysis. One of the limitations of this study is publication bias, which was not assessed because the numbers of trials reporting OS, PFS, ORR, and AEs were <10. Second, our analysis is pooled from summary data rather than data from the individual patients from each study. Third, although there was a statistical difference of OS between bevacizumab plus platinum-based chemotherapy and chemotherapy alone in race (white), smoking status (past), bodyweight loss (≤5%), and histology (adenocarcinoma), the results were only extracted from one study. Thus, more studies in this subject might demonstrate the real relationship between bevacizumab plus platinum-based chemotherapy and chemotherapy alone in terms of OS based on these subgroups. Finally, there was a statistical difference of PFS between bevacizumab plus platinum-based chemotherapy and chemotherapy alone in histology (large cell), but the results were only extracted from one study. Therefore, more studies in this subject might demonstrate the real relationship between bevacizumab plus platinum-based chemotherapy and chemotherapy alone in terms of PFS based on these subgroups. However, the risk of publication is inherent in meta-analyses, and we believe that our result is convincing. More RCTs of high quality are required in future work.

## Conclusions

This meta-analysis demonstrated that bevacizumab significantly increased the OS, extended the PFS, and improved the ORR when added to first-line platinum-based chemotherapy in patients with advanced NSCLC, with no new safety signals found. Stratification analysis based on treatment and bevacizumab dose showed that only the higher dose (15 mg/kg) of bevacizumab plus CP significantly extended the OS, and PFS, but both 7.5 mg/kg and 15 mg/kg of bevacizumab improved ORR. However, both 7.5 mg/kg and 15 mg/kg of bevacizumab could only increase PFS and ORR, but not extend OS, when added to CG.

## Data Availability Statement

The original contributions presented in the study are included in the article/Supplementary Material, further inquiries can be directed to the corresponding authors.

## Author Contributions

H-ML and RW designed the study. H-ML and YL wrote the manuscript and performed the statistical analysis of the data. YL revised and polished the manuscript. All authors contributed to the article and approved the submitted version.

## Conflict of Interest

The authors declare that the research was conducted in the absence of any commercial or financial relationships that could be construed as a potential conflict of interest.

## Abbreviations

NSCLC: non-small-cell lung cancer
RCTs: randomized controlled trials
RR: risk ratio
BCP: bevacizumab-carboplatin-paclitaxel
BCG: bevacizumab-cisplatin-gemcitabine
OS: overall survival
PFS: progression-free survival
ORR: objective response rate
AEs: adverse events
HRs: hazard ratios.

## References

[1] Ries L Melbert D Krapcho M Stinchcomb D Howlader N Horner M. SEER Cancer Statistics Review, 1975-2006, National Cancer Institute. Bethesda, MD: My Publications (2006).

[2] Soria JC Mauguen A Reck M Sandler AB Saijo N Johnson DH. Systematic review and meta-analysis of randomised, phase II/III trials adding bevacizumab to platinum-based chemotherapy as first-line treatment in patients with advanced non-small-cell lung cancer. Ann Oncol. (2013) 24:20–30. 10.1093/annonc/mds590 23180113

[3] Gridelli C Maione P Rossi A De Marinis F. The role of bevacizumab in the treatment of non-small cell lung cancer: current indications and future developments. Oncologist. (2007) 12:1183–93. 10.1634/theoncologist.12-10-1183 17962612

[4] Bearz A Serraino D Fratino L Berretta M Tirelli U. Recent improvement in the survival of patients with advanced nonsmall cell lung cancer enrolled in phase III trials of first-line, systemic chemotherapy. Cancer. (2007) 110:2593–94. 10.1002/cncr.23063 17960771

[5] Soon YY Stockler MR Askie LM Boyer MJ. Duration of chemotherapy for advanced non-small-cell lung cancer: a systematic review and meta-analysis of randomized trials. J Clin Oncol. (2009) 27:3277–83. 10.1200/JCO.2008.19.4522 19470938

[6] Planchard D Popat S Kerr K Novello S Smit EF Faivre-Finn C. Metastatic non-small cell lung cancer: ESMO clinical practice guidelines for diagnosis, treatment and follow-up. Ann Oncol. (2018) 29:iv192–237. 10.1093/annonc/mdy275 30285222

[7] Ferrara N Hillan KJ Gerber HP Novotny W. Discovery and development of bevacizumab, an anti-VEGF antibody for treating cancer. Nat Rev Drug Discov. (2004) 3:391–400. 10.1038/nrd1381 15136787

[8] Sandler A Gray R Perry MC Brahmer J Schiller JH Dowlati A. Paclitaxel-carboplatin alone or with bevacizumab for non-small-cell lung cancer. N Engl J Med. (2006) 355:2542–50. 10.1056/NEJMoa061884 17167137

[9] Sandler A Yi J Dahlberg S Kolb MM Wang L Hambleton J. Treatment outcomes by tumor histology in eastern cooperative group study E4599 of bevacizumab with paclitaxel/carboplatin for advanced non-small cell lung cancer. J Thorac Oncol. (2010) 5:1416–23. 10.1097/JTO.0b013e3181da36f4 20686429

[10] Zhou C Wu YL Chen G Liu X Zhu Y Lu S. BEYOND: a randomized, double-blind, placebo-controlled, multicenter, Phase III study of first-line carboplatin/paclitaxel plus bevacizumab or placebo in chinese patients with advanced or recurrent nonsquamous non-small-cell lung cancer. J Clin Oncol. (2015) 33:2197–204. 10.1200/JCO.2014.59.4424 26014294

[11] Ramalingam SS Dahlberg SE Langer CJ Gray R Belani CP Brahmer JR. Outcomes for elderly, advanced-stage non small-cell lung cancer patients treated with bevacizumab in combination with carboplatin and paclitaxel: analysis of eastern cooperative oncology group trial 4599. J Clin Oncol. (2008) 26:60–65. 10.1200/JCO.2007.13.1144 18165641

[12] Lopez-Chavez A Young T Fages S Leon L Schiller JH Dowlati A. Bevacizumab maintenance in patients with advanced non-small-cell lung cancer, clinical patterns, and outcomes in the eastern cooperative oncology group 4599 study: results of an exploratory analysis. J Thorac Oncol. (2012) 7:1707–12. 10.1097/JTO.0b013e318265b500 23059774

[13] Niho S Kunitoh H Nokihara H Horai T Ichinose Y Hida T. Randomized phase II study of first-line carboplatin-paclitaxel with or without bevacizumab in Japanese patients with advanced non-squamous non-small-cell lung cancer. Lung Cancer. (2012) 76:362–7. 10.1016/j.lungcan.2011.12.005 22244743

[14] Reck M Von Pawel J Zatloukal P Ramlau R Gorbounova V Hirsh V. Phase III trial of cisplatin plus gemcitabine with either placebo or bevacizumab as first-line therapy for nonsquamous non-small-cell lung cancer: AVAil. J Clin Oncol. (2009) 27:1227–34. 10.1200/JCO.2007.14.5466 19188680

[15] Yang K Wang YJ Chen XR Chen HN. Effectiveness and safety of bevacizumab for unresectable non-small-cell lung cancer: a meta-analysis. Clin Drug Investig. (2010) 30:229–41. 10.2165/11532260-000000000-00000 20225906

[16] Botrel TE Clark O Clark L Paladini L Faleiros E Pegoretti B. Efficacy of bevacizumab (Bev) plus chemotherapy (CT) compared to CT alone in previously untreated locally advanced or metastatic non-small cell lung cancer (NSCLC): systematic review and meta-analysis. Lung Cancer. (2011) 74:89–97. 10.1016/j.lungcan.2011.01.028 21377753

[17] Reck M Von Pawel J Zatloukal P Ramlau R Gorbounova V Hirsh V. Overall survival with cisplatin-gemcitabine and bevacizumab or placebo as first-line therapy for nonsquamous non-small-cell lung cancer: results from a randomised phase III trial (AVAiL). Ann Oncol. (2010) 21:1804–9. 10.1093/annonc/mdq020 20150572 PMC2924992

[18] Higgins JPT Green S editors. Cochrane Handbook for Systematic Reviews of Interventions Version 5.1.0. The Cochrane Collaboration (2011). Available online at: www.handbook.cochrane.org

[19] Liberati A Altman DG Tetzlaff J Mulrow C Gøtzsche PC Ioannidis JP. The PRISMA statement for reporting systematic reviews and meta-analyses of studies that evaluate health care interventions: explanation and elaboration. J Clin Epidemiol. (2009) 62:e1–34. 10.1016/j.jclinepi.2009.06.006 19631507

[20] Li HM Zhang RJ Gao H Jia CY Zhang JX Dong FL. New vertebral fractures after osteoporotic vertebral compression fracture between balloon kyphoplasty and nonsurgical treatment PRISMA. Medicine. (2018) 97:e12666. 10.1097/MD.0000000000012666 30290650 PMC6200511

[21] Li H-M Zhang R-J Shen C-L. Differences in radiographic and clinical outcomes of oblique lateral interbody fusion and lateral lumbar interbody fusion for degenerative lumbar disease: a meta-analysis. BMC Muscul Disord. (2019) 20:582. 10.1186/s12891-019-2972-7 31801508 PMC6894220

[22] Li HM Zhang RJ Shen CL. Accuracy of pedicle screw placement and clinical outcomes of robot-assisted technique versus conventional freehand technique in spine surgery from nine randomized controlled trials: a meta-analysis. Spine. (2020) 45:E111–9. 10.1097/BRS.0000000000003193 31404053

[23] Li HM Liu Y Zhang RJ Ding JY Shen CL. Vitamin D receptor gene polymorphisms and osteoarthritis: a meta-analysis. Rheumatology. (2021) 60:538–48. 10.1093/rheumatology/keaa644 33147632

[24] Li HM Zhang RJ Shen CL. Radiographic and clinical outcomes of oblique lateral interbody fusion versus minimally invasive transforaminal lumbar interbody fusion for degener ative lumbar disease. World Neurosurg. (2019) 122:e627–38. 10.1016/j.wneu.2018.10.115 31108079

[25] Johnson DH Fehrenbacher L Novotny WF Herbst RS Nemunaitis JJ Jablons DM. Randomized phase II trial comparing bevacizumab plus carboplatin and paclitaxel with carboplatin and paclitaxel alone in previously untreated locally advanced or metastatic non-small-cell lung cancer. J Clin Oncol. (2004) 22:2184–91. 10.1200/JCO.2004.11.022 15169807

[26] Hurwitz H Fehrenbacher L Novotny W Cartwright T Hainsworth J Heim W. Bevacizumab plus irinotecan, fluorouracil, and leucovorin for metastatic colorectal cancer. N Engl J Med. (2004) 350:2335–42. 10.1056/NEJMoa032691 15175435

[27] Zielinski C Láng I Inbar M Kahán Z Greil R Beslija S. Bevacizumab plus paclitaxel versus bevacizumab plus capecitabine as first-line treatment for HER2-negative metastatic breast cancer (TURANDOT): primary endpoint results of a randomised, open-label, non-inferiority, phase 3 trial. Lancet Oncol. (2016) 17:1230–39. 10.1016/S1470-2045(16)30154-1 27501767

[28] Hainsworth JD Sosman JA Spigel DR Edwards DL Baughman C Greco A. Treatment of metastatic renal cell carcinoma with a combination of bevacizumab and erlotinib. J Clin Oncol. (2005) 23:7889–96. 10.1200/JCO.2005.01.8234 16204015

[29] Soria JC Massard C Le Chevalier T. Should progression-free survival be the primary measure of efficacy for advanced NSCLC therapy? Ann Oncol. (2010) 21:2324–32. 10.1093/annonc/mdq204 20497965

[30] Sandler AB Schiller JH Gray R Dimery I Brahmer J Samant M. Retrospective evaluation of the clinical and radiographic risk factors associated with severe pulmonary hemorrhage in first-line advanced, unresectable non-small-cell lung cancer treated with Carboplatin and Paclitaxel plus bevacizumab. J Clin Oncol. (2009) 27:1405–12. 10.1200/JCO.2008.16.2412 19224857 PMC3527732

[31] Laskin J Crinò L Felip E Franke F Gorbunova V Groen H. Safety and efficacy of first-line bevacizumab plus chemotherapy in elderly patients with advanced or recurrent nonsquamous non-small cell lung cancer: safety of avastin in lung trial (MO19390). J Thorac Oncol. (2012) 7:203–11. 10.1097/JTO.0b013e3182370e02 22173662

