# Supplementary material for: Effectiveness and Safety of Adding Bevacizumab to Platinum-Based Chemotherapy as First-Line Treatment for Advanced Non-Small-Cell Lung Cancer: A Meta-Analysis
Source: Front Med (Lausanne). 2021 Jun 30;8:616380. doi: 10.3389/fmed.2021.616380 (PMC8277997; doi:10.3389/fmed.2021.616380)
Supplement: Supplementary file 1 [file Table_1.DOCX]

eTable 1. Search Strategy for Each Database

| Database | Search strategy |
| --- | --- |
| Pubmed | #1 "bevacizumab"[MeSH Terms] OR "bevacizumab"[All Fields] OR "bevacizumab s"[All Fields] OR "bevacizumab"[MeSH Terms] OR "bevacizumab"[All Fields] OR "avastin"[All Fields] OR "bevacizumab s"[All Fields]  #2 "lung neoplasms"[MeSH Terms] OR ("lung neoplasms"[MeSH Terms] OR ("lung"[All Fields] AND "neoplasms"[All Fields]) OR "lung neoplasms"[All Fields] OR ("lung neoplasms"[MeSH Terms] OR ("lung"[All Fields] AND "neoplasms"[All Fields]) OR "lung neoplasms"[All Fields] OR ("pulmonary"[All Fields] AND "neoplasms"[All Fields]) OR "pulmonary neoplasms"[All Fields]) OR ("lung neoplasms"[MeSH Terms] OR ("lung"[All Fields] AND "neoplasms"[All Fields]) OR "lung neoplasms"[All Fields] OR ("neoplasms"[All Fields] AND "lung"[All Fields]) OR "neoplasms lung"[All Fields]) OR ("lung neoplasms"[MeSH Terms] OR ("lung"[All Fields] AND "neoplasms"[All Fields]) OR "lung neoplasms"[All Fields] OR ("lung"[All Fields] AND "neoplasm"[All Fields]) OR "lung neoplasm"[All Fields]) OR ("lung neoplasms"[MeSH Terms] OR ("lung"[All Fields] AND "neoplasms"[All Fields]) OR "lung neoplasms"[All Fields] OR ("neoplasm"[All Fields] AND "lung"[All Fields]) OR "neoplasm lung"[All Fields]) OR ("lung neoplasms"[MeSH Terms] OR ("lung"[All Fields] AND "neoplasms"[All Fields]) OR "lung neoplasms"[All Fields] OR ("neoplasms"[All Fields] AND "pulmonary"[All Fields]) OR "neoplasms pulmonary"[All Fields]) OR ("lung neoplasms"[MeSH Terms] OR ("lung"[All Fields] AND "neoplasms"[All Fields]) OR "lung neoplasms"[All Fields] OR ("neoplasm"[All Fields] AND "pulmonary"[All Fields]) OR "neoplasm pulmonary"[All Fields]) OR ("lung neoplasms"[MeSH Terms] OR ("lung"[All Fields] AND "neoplasms"[All Fields]) OR "lung neoplasms"[All Fields] OR ("pulmonary"[All Fields] AND "neoplasm"[All Fields]) OR "pulmonary neoplasm"[All Fields]) OR ("lung neoplasms"[MeSH Terms] OR ("lung"[All Fields] AND "neoplasms"[All Fields]) OR "lung neoplasms"[All Fields] OR ("lung"[All Fields] AND "cancer"[All Fields]) OR "lung cancer"[All Fields]) OR ("lung neoplasms"[MeSH Terms] OR ("lung"[All Fields] AND "neoplasms"[All Fields]) OR "lung neoplasms"[All Fields] OR ("cancer"[All Fields] AND "lung"[All Fields]) OR "cancer lung"[All Fields]) OR ("lung neoplasms"[MeSH Terms] OR ("lung"[All Fields] AND "neoplasms"[All Fields]) OR "lung neoplasms"[All Fields] OR ("cancers"[All Fields] AND "lung"[All Fields]) OR "cancers lung"[All Fields]) OR ("lung neoplasms"[MeSH Terms] OR ("lung"[All Fields] AND "neoplasms"[All Fields]) OR "lung neoplasms"[All Fields] OR ("lung"[All Fields] AND "cancers"[All Fields]) OR "lung cancers"[All Fields]) OR ("lung neoplasms"[MeSH Terms] OR ("lung"[All Fields] AND "neoplasms"[All Fields]) OR "lung neoplasms"[All Fields] OR ("pulmonary"[All Fields] AND "cancer"[All Fields]) OR "pulmonary cancer"[All Fields]) OR ("lung neoplasms"[MeSH Terms] OR ("lung"[All Fields] AND "neoplasms"[All Fields]) OR "lung neoplasms"[All Fields] OR ("cancer"[All Fields] AND "pulmonary"[All Fields]) OR "cancer pulmonary"[All Fields]) OR ("lung neoplasms"[MeSH Terms] OR ("lung"[All Fields] AND "neoplasms"[All Fields]) OR "lung neoplasms"[All Fields] OR ("cancers"[All Fields] AND "pulmonary"[All Fields]) OR "cancers pulmonary"[All Fields]) OR ("lung neoplasms"[MeSH Terms] OR ("lung"[All Fields] AND "neoplasms"[All Fields]) OR "lung neoplasms"[All Fields] OR ("pulmonary"[All Fields] AND "cancers"[All Fields]) OR "pulmonary cancers"[All Fields]) OR ("lung neoplasms"[MeSH Terms] OR ("lung"[All Fields] AND "neoplasms"[All Fields]) OR "lung neoplasms"[All Fields] OR ("cancer"[All Fields] AND "lung"[All Fields]) OR "cancer of the lung"[All Fields]) OR ("lung neoplasms"[MeSH Terms] OR ("lung"[All Fields] AND "neoplasms"[All Fields]) OR "lung neoplasms"[All Fields] OR ("cancer"[All Fields] AND "lung"[All Fields]) OR "cancer of lung"[All Fields]))  #3 "randomized controlled trial"[Publication Type] OR "randomized controlled trials as topic"[MeSH Terms] OR "randomized controlled trial"[All Fields] OR "randomised controlled trial"[All Fields]  #4 #1 AND #2 AND #3 |
| Embase | #1 'bevacizumab'/exp OR bevacizumab OR 'avastin'/exp OR avastin  #2 'randomized controlled trial'/exp OR 'randomized controlled trial'  #3 'lung neoplasms'/exp OR 'lung neoplasms' OR (('lung'/exp OR lung) AND ('neoplasms'/exp OR neoplasms)) OR 'neoplasms pulmonary' OR (('neoplasms'/exp OR neoplasms) AND pulmonary) OR 'pulmonary cancers' OR (pulmonary AND ('cancers'/exp OR cancers)) OR 'cancer of lung' OR (('cancer'/exp OR cancer) AND of AND ('lung'/exp OR lung))  #4 #1 AND #2 AND #3 |
| Cochrane library | #1 (avastin):ti,ab,kw OR (avastin):ti,ab,kw (Word variations have been searched)  #2 (lung neoplasms):ti,ab,kw OR (pulmonary cancers):ti,ab,kw OR (cancer of lung):ti,ab,kw (Word variations have been searched)  #3 randomized controlled trial (Word variations have been searched)  #4 #1 AND #2 AND #3 |
