# Supplementary material for: Effectiveness and Safety of Adding Bevacizumab to Platinum-Based Chemotherapy as First-Line Treatment for Advanced Non-Small-Cell Lung Cancer: A Meta-Analysis
Source: Front Med (Lausanne). 2021 Jun 30;8:616380. doi: 10.3389/fmed.2021.616380 (PMC8277997; doi:10.3389/fmed.2021.616380)
Supplement: Supplementary file 2 [file Table_2.DOCX]

eTable 2. Subgroup analyses of OS based on patient characteristics.

| Category | | No. of  studies | Tests of association | | | Tests of heterogeneity | |
| --- | --- | --- | --- | --- | --- | --- | --- |
|  |  |  | HR | 95%CI | *P*-value | I^2^, % | *P*-value |
| Sex | Male | 2 | 0.70 | 0.58,0.84 | **0.000** | 0.0% | 0.896 |
|  | Female | 2 | 0.82 | 0.53,1.27 | 0.378 | 62.4% | 0.103 |
| Age | < 65 | 2 | 0.68 | 0.57,0.81 | **0.000** | 0.0% | 0.415 |
|  | ≥ 65 | 2 | 0.90 | 0.71,1.13 | 0.357 | 0.0% | 0.832 |
| Stage | III B | 2 | 0.71 | 0.44,1.12 | 0.139 | 0.0% | 0.622 |
|  | IV non recurrent | 2 | 0.78 | 0.60,1.02 | 0.069 | 54.4% | 0.139 |
|  | Recurrent | 1 | 0.66 | 0.38,1.14 |  |  |  |
| Race | White | 1 | 0.83 | 0.7,0.98 |  |  |  |
|  | Black | 1 | 0.46 | 0.21,1.03 |  |  |  |
|  | Other | 1 | 1.92 | 0.37,9.97 |  |  |  |
| Bodyweight loss | ≤5% | 1 | 0.77 | 0.64,0.92 |  |  |  |
|  | >5% | 1 | 0.85 | 0.63,1.14 |  |  |  |
| Smoking status | Current | 1 | 0.76 | 0.5,1.16 |  |  |  |
|  | Past | 1 | 0.56 | 0.36,0.87 |  |  |  |
| Histology | Adenocarcinoma | 1 | 0.69 | 0.58,0.83 |  |  |  |
|  | Large cell | 1 | 1.15 | 0.6,2.24 |  |  |  |
|  | NSCLC, NOS | 1 | 1.16 | 0.84,1.61 |  |  |  |
|  | Other | 1 | 0.92 | 0.43,1.98 |  |  |  |

HR, hazard ratio; CI, confidence intervals. Bold values indicate *P* < 0.05.
