# Supplementary material for: Effectiveness and Safety of Adding Bevacizumab to Platinum-Based Chemotherapy as First-Line Treatment for Advanced Non-Small-Cell Lung Cancer: A Meta-Analysis
Source: Front Med (Lausanne). 2021 Jun 30;8:616380. doi: 10.3389/fmed.2021.616380 (PMC8277997; doi:10.3389/fmed.2021.616380)
Supplement: Supplementary file 3 [file Table_3.DOCX]

eTable 3. Subgroup analyses of PFS based on patient characteristics.

| Category | | No. of  studies | Tests of association | | | Tests of heterogeneity | |
| --- | --- | --- | --- | --- | --- | --- | --- |
|  |  |  | HR | 95%CI | *P*-value | I^2^, % | *P*-value |
| Sex | Male | 2 | 0.45 | 0.33,0.60 | **0.000** | 0.0% | 0.336 |
|  | Female | 2 | 0.45 | 0.30,0.65 | **0.000** | 3.0% | 0.310 |
| Age | < 65 | 2 | 0.39 | 0.30,0.51 | **0.000** | 0.0% | 0.400 |
|  | ≥ 65 | 2 | 0.59 | 0.38,0.91 | **0.018** | 0.0% | 0.358 |
| Stage | III B | 2 | 0.39 | 0.20,0.76 | **0.005** | 0.0% | 0.596 |
|  | IV non recurrent | 2 | 0.50 | 0.27,0.91 | **0.025** | 81.2% | **0.021** |
|  | Recurrent | 2 | 0.24 | 0.07,0.85 | **0.027** | 0.0% | 0.825 |
| Smoking status | Current | 1 | 0.44 | 0.28,0.69 |  |  |  |
|  | Past | 2 | 0.45 | 0.26,0.75 | **0.002** | 67.5% | 0.080 |
|  | Never | 1 | 0.49 | 0.24,1.01 |  |  |  |
| Histology | Adenocarcinoma | 1 | 0.65 | 0.54,0.78 |  |  |  |
|  | Large cell | 1 | 0.35 | 0.15,0.79 |  |  |  |
|  | NSCLC, NOS | 1 | 0.78 | 0.55,1.09 |  |  |  |
|  | Other | 1 | 0.54 | 0.19,1.52 |  |  |  |

HR, hazard ratio; CI, confidence intervals. Bold values indicate *P* < 0.05.
